# Laboratory study on reattachment of vertical root fractures using 4-META/MMA-TBB resin

**DOI:** 10.3389/fdmed.2025.1593189

**Published:** 2025-06-27

**Authors:** Huiying Li, Tong Wang, Jing Fu, Jinghan Guo, Zhimin Fang, Keqing Pan, Haiping Xu

**Affiliations:** ^1^ Department of Stomatology, The Affiliated Hospital of Qingdao University, Qingdao, China; ^2^ School of Stomatology, Qingdao University, Qingdao, China; ^3^ Information Management Department, The Affiliated Hospital of Qingdao University, Qingdao, China

**Keywords:** vertical root fracture, root fragment reattachment, 4-META/MMA-TBB resin, biocompatibility, fracture resistance

## Abstract

**Background:**

Reattachment of root fragments with appropriate adhesive materials is expected to be the last conservative treatment for preserving teeth with vertical root fractures (VRFs).

**Objective:**

This study evaluated the biocompatibility of 4-META/MMA-TBB resin for root repair, compared with iRoot BP Plus, Fuji IX GIC, and Clearfil SA Luting. Fracture resistance and microleakage of the reattached roots were also tested.

**Methods:**

The biocompatibility of set materials was evaluated on L929 fibroblasts. Cell Counting Kit-8 (CCK-8) assay, live/dead cells staining and flow cytometry were used to assess cell biocompatibility. VRFs were created in bovine teeth, which were then reattached with set materials (excluding iRoot BP Plus). For the fracture resistance test, the roots were vertically fractured through the root canals (*n* = 20). The fracture resistance was compared with sound roots (control group) and fracture patterns were observed under a microscope. Microleakage was also tested on the reattached roots (*n* = 10). Results were analyzed by one-way ANOVA and the Tukey test. The significance level was set at *α* = 0.05.

**Results:**

Clearfil SA Luting group exhibited the highest cytotoxicity. The other test materials had acceptable cytotoxicity, not exceeding Grade 1 [relative growth ratio (RGR) > 75%] in CCK-8. Flow cytometry showed that the proportion of viable cells exposed to 4-META/MMA-TBB resin displayed no significant difference compared with iRoot BP Plus (*P* > 0.05). The root fracture resistances reattached using 4-META/MMA-TBB resin and Clearfil SA Luting were higher than that by Fuji IX GIC, but lower than those of the control group (*P* < 0.05). The difference between the two resin groups was statistically insignificant (*P* > 0.05). As for the microleakage, 4-META/MMA-TBB resin group had the shortest penetration depth, whereas Fuji IX GIC group showed the longest penetration (*P* < 0.05).

**Conclusions:**

4-META/MMA-TBB resin had acceptable cell biocompatibility for root repair, similar to iRoot BP Plus. It can provide good fracture resistance and excellent sealing effect for reattaching treatment of VRFs.

## 1 Introduction

Vertical root fracture (VRF), characterized by a longitudinal crack within the root, is particularly prevalent (86.79%) in people over 40 years old (1). Due to its poor prognosis, VRF constitutes a major challenge for endodontists and has been reported to account for up to 31.7% of tooth loss (2). Treating and therefore preserving a vertically fractured tooth contributes to improving both masticatory function and aesthetics, while retaining the integrity of the natural dentition increases healthy life expectancy (3). In addition, considering the complications associated with dental implants (4), tooth preservation can be selected in appropriate cases when the patient desires to conserve the fractured tooth. Therefore, reattachment of vertically fractured fragments with adhesive cement, as a conservative method, has demonstrated short-term success with proper management (5, 6). Periodontal healing was observed clinically 22–33 months after bonding treatment, such as decreased probing depth, bleeding score, and tooth mobility, and increased alveolar bone height (5).

Reattachment of root fragments has been performed via the root canal, via the external root surface under gingival flap operation, or *in vitro* combined with intentional replantation (3). For intraoral approaches, it's hard to reach the entire fracture line without tooth extraction, leading to the persistence of some bacteria there. To completely prepare the fracture surfaces, additional alveolar bone must be removed during flapping, which is not conducive to the healing of periodontal tissue (7). Therefore, extraoral bonding followed by intentional replantation seems to be an ideal alternative treatment option (6, 8). In order to improve treatment success, basic researches have evaluated the impact of surgical procedures, pretreatment of bonding interface, the application of fiber posts, and bone defects on treatment (7, 9–11). However, the success of the treatment might rely more on the adhesive materials.

In recent years, a variety of materials have been explored for applications in the reattaching treatment of fractured roots. Glass ionomer cements (GIC) were used previously for their self-adhesive property and fluoride release (12). But neither the bonding ability nor the material strength is strong enough to hold the fractured root fragments (13). Adhesive resin cements, with excellent mechanical properties, had been reported to potentially offer increased fracture resistance for VRF affected roots (14). But the elution of toxic monomers, as a result of a low conversion degree, would be a potential problem for the periodontal tissue of teeth (15). Calcium silicate-based cements, such as MTA, biodentine or iRoot BP plus, are indicated for various root repairs, including root fracture sealing due to the high bioactivity for periodontal tissue (16–18). But calcium silicate-based materials cannot provide enough bonding strength to dentin (19). A novel dual-layered repairing approach, which uses composite resin as adhesive cement and is covered with a layer of calcium silicate-based materials in contact the periodontal tissue, has been developed (7). This design attempted to balance bonding strength and biocompatibility. However, calcium silicate-based materials could occupy part of the limited adhesive area otherwise available for resin bonding, potentially reducing bond force. These factors may limit their broader clinical application. Recently, 4-methacryloxyethyl trimellitate anhydride/methyl methacrylate-tri-n-butyl borane (4-META/MMA-TBB) resin has been successfully used in the treatment of VRF in several clinical cases (6, 8, 20). The material was reported to adhere well to dentin and polymerize highly under wet conditions, offering good biocompatibility (21–23). The treatment success rate was reported to be 88.5% at 1 year and 59.3% at 5 years on 26 cases (24). However, 4-META/MMA-TBB resin was initially designed to be applied to the bonding of tooth crowns. Preliminary studies on root fragment reattachment were mainly based on clinical cases, and the biological and mechanical research on the materials still needs to be explored in the laboratory.

An ideal material for reattaching fractured tooth roots should possess excellent biocompatibility, provide high bonding strength, and exhibit less microleakage. Since the bonding material comes into direct contact with the periodontal tissue, biocompatibility of the material can affect the cells, potentially triggering gingival inflammation and ultimately leading to treatment failure (25). Moreover, the bonding performance of the adhesive is of particular importance. As teeth are constantly subjected to masticatory forces, adhesive materials should have sufficiently high bonding strength, which is a crucial requirement for the long-term success of root repair (26). It is also crucial that the bonding material effectively prevents microleakage at the fracture line. Microleakage could increase the risk of bacterial and their byproduct leakage between the root canal and periodontal tissue, ultimately leading to the failure of treatment (27).

In this study, the biocompatibility of 4-META/MMA-TBB resin for root repair was evaluated by Cell Counting Kit-8 (CCK-8) assay, live/dead fluorescence staining and flow cytometry, compared with iRoot BP Plus, Fuji IX GIC, and Clearfil SA Luting. Microleakage and fracture tests were performed on reattached roots to evaluate the effectiveness and strength of the adhesion. The null hypothesis is that there will be no difference in terms of biocompatibility, microleakage and fracture resistance on reattached teeth using different adhesive materials.

## 2 Materials and methods

### 2.1 Biocompatibility

#### 2.1.1 Specimen and extract preparation

Four commercial cement materials used in reattachment of root fracture fragments were tested in this study (Table 1). Cylindric specimens (diameter: 10 mm; height: 1 mm) of each material were prepared in plastic molds after thoroughly mixing. Specimens of Clearfil SA Luting were light-cured following the manufacturer's instructions using 500 mW/cm^2^ curing lamps (Kerr, Demiplus, CA, USA). 4-META/MMA-TBB resin, iRoot BP Plus and Fuji IX GIC specimens were stored at 37℃, 100% humidity for 3 days. After complete solidification, specimens were polished in sequence with 1,000 and 4,000 grit SiC paper (MATADOR, Remscheid, Germany) to standardize the sample surface area.

**Table 1:** Materials used in this study.

Materials	Manufacturers	Component
Super-bond C&B (4-META/MMA-TBB resin)	Sun Medical, Moriyama, Japan	Liquid:(monomer) MMA, 4-META
		(catalyst) Tri-n-butyl borane
		Powder: PMMA
iRoot BP Plus	Innovative, Bioceramix Inc, Canada	Tricalcium silicates, dicalcium silicates, zirconium oxide, tantalum pentoxide, calcium phosphate monobasic
Fuji IX GP	GC, Tokyo, Japan	Liquid: polyacrylic acid, polycarboxylic acid
		Powder: fluoroaluminosilicate glass, polyacrylic acid
Clearfil SA Luting	Kuraray medical, Tokyo, Japan	Bis-GMA, TEGDMA, MDP, barium glass, silica, sodium fluoride

For sterilization, each surface of the sample was exposed to ultraviolet light for 30 min. According to ISO 10993-12, the sterilized samples were immersed in DMEM (Procell, Wuhan, China) with 10% (v/v) fetal bovine serum (Procell, Wuhan, China) and 1% (v/v) penicillin/streptomycin (Procell, Wuhan, China) at a surface/volume ratio of 3 cm^2^/ml in a 37℃ incubator for 24 h. The solution was collected and filtered through a 0.22-micron microfilter to obtain test extract.

#### 2.1.2 Cell culture

Mouse L929 fibroblasts at passage P3 to P5 were incubated in complete culture medium at 37℃ with 5% CO_2_ in the humidified incubator. Cells were trypsinized for 3 min to prepare a single-cell suspension, which was then diluted and seeded in multi-well plates at a density of 2 × 10^3^ cells per well for CCK-8 or 1 × 10^4^ cells per well for other tests. After 24 h incubation for complete adherence, the cultures were replaced with different extracts (four experimental groups) or complete culture medium (negative control), with five replicates per group. After different time of treatment (specified bellow), the cultures were removed and the adherent cells were subjected to subsequent tests.

#### 2.1.3 CCK-8 assay

After treatment in 96-well plate for 1, 2, 3 and 5days, the medium was replaced with 90 μl of complete medium and 10μl of CCK-8 solution (Elabscience, Wuhan, China) per well. The plates were then incubated at 37℃ for 1 h. The OD value of each group was measured at a wavelength of 450 nm with a microplate reader (Spectra Max iD3, Sunnyvale, CA, Molecular Devices). The relative growth ratio (RGR) of the cells was calculated as follows:

(1) $$\mathrm{RGR}\;(\text{\%})=\frac{OD_{\mathrm{experimental}}}{OD_{\mathrm{control}}}\times 100\text{\%},$$

The cytotoxicity in each group was graded as shown in Table 2 (28).

**Table 2:** Cytotoxicity grading standard.

RGR	Cytotoxicity classification	Cytotoxicity
≥100%	0	-
75%–99%	1	-
50%–74%	2	Mild
25%–49%	3	Moderate
1%–24%	4	Severe
0	5	Severe

#### 2.1.4 Live/dead cell staining

After 1, 2 and 3 days of treatment in 24-well plate, the medium was removed. Cells were washed twice with PBS (Beyotime, Shanghai, China) and stained with Calcein AM/PI Cell Vitality/Cytotoxicity Assay (Beyotime, Shanghai, China) at room temperature in the dark for 20 min. Images were captured using a fluorescence microscope (Eclipse Ti-E, Nikon, Tokyo, Japan) and merged with Image J (Bethesda, Montgomery County, MD, USA). Live cells emitted green fluorescence at 494 nm, and dead cells emitted red at 523 nm.

#### 2.1.5 Flow cytometry

Cells were treated in 6-well plates for 2 days. The adherent cells were detached using trypsin-EDTA, mixed with floating cells, and centrifuged at 1,200 rpm for 5 min at 4℃ to discard the supernatant. The cell pellet was resuspended in binding buffer with a concentration of 1 × 10^4 ^cells/ml. Then 2.5 μl of Annexin V-FITC and 2.5 μl of PI were added to the cell suspension according to the manufacturer's (Beyotime, Shanghai, China) instructions. The mixture was gently vortexed and incubated in the dark for 30 min. The stained cells were analyzed on a flow cytometer (Accuri C6 Plus, BD Biosciences, Massachusetts, USA), using FlowJo-V10 (Tree Star, Woodburn, USA).

### 2.2 Fracture and microleakage test

#### 2.2.1 Specimen preparation

Freshly extracted bovine incisors from 7-year-old cattle were collected and meticulously debrided of calculus and soft tissues. A dental microscope (M320, Leica Microsystems, Germany) was used to observe any root cracks or open apices, and the respective teeth were excluded. The selected teeth were stored in Hank's solution at 4°C and utilized within 1 month. The utilization of these bovine teeth was approved by the Ethics Committee of the Affiliated Hospital of Qingdao University (Ethical approval number: QYFYWZLL29577). The crowns were removed, leaving 15 mm roots. The external diameters of the roots were precisely recorded in buccolingual and mesiodistal direction to make sure the selected roots had uniform size (Table 3). The root canal diameter at the top was less than 2 mm initially, and was prepared into a uniform size of 3 mm using a tapered file (Lionwell, Jiangsu, Taicang, China).

**Table 3:** The buccolingual and mesiodistal root diameter(mm), fracture resistance(N), fracture patterns and penetration depth(mm) of different groups.

Groups	Buccolingual	Mesiodistal	Fracture resistance	Type-II	Type-III	Penetration depth
Control	7.83 ± 0.46^#^	6.44 ± 0.45*	278.50 ± 45.19^a^	/		
4-META/MMA- TBB resin	7.83 ± 0.36^#^	6.59 ± 0.51*	220.90 ± 45.01^b^	2	18	0.63 ± 0.15^α^
Fuji IX GIC	7.73 ± 0.43^#^	6.63 ± 0.55*	53.16 ± 15.76^c^	0	20	2.08 ± 0.17^β^
Clearfil SA Luting	7.57 ± 0.47^#^	6.19 ± 0.68*	184.00 ± 45.45^b^	1	19	1.44 ± 0.19^γ^

The same symbols in each column indicate no statistical difference between groups (*P* > 0.05).

^a,b,c^are used for fracture resistance.

^#,*^are used for buccolingual diameter and mesiodistal diameter.

^α,β,γ^are used for microleakage.

Specimens were randomly allocated into 4 groups (*n* = 20). In the control group, the root canals were directly filled with gutta-percha. In the experimental groups, VRFs were created and reattached with 4-META/MMA-TBB resin, Fuji IX GIC, or Clearfil SA Luting. iRoot BP Plus was excluded due to poor bonding.

To produce a VRF, a 3 mm depth notch was cut into the coronal sections of the root buccolingually using a diamond disc (IsoMetTM, Buehler, Lake Bluff, IL, USA), and the root was split vertically. Bonding surfaces were prepared by ultrasound. A size 3,004 gutta-percha point was placed in the canal to facilitate loading. Specimens were incubated at 37°C for 24 h to allow material solidify.

#### 2.2.2 Fracture test

Roots were wrapped with a 0.2 mm thick silicone rubber material to mimic the periodontal ligament, and embedded in self-curing resin blocks, leaving 2 mm of the tooth neck exposed. The specimens were fixed on the lower plate of a universal testing machine (Instron, Norwood, MA, USA). A conical steel pin with a diameter of 1.5 mm was connected to the upper plate and lowered to make contact with the gutta-percha inside the root canal. A vertical load was applied at a speed of 1 mm/min until fracture. The fracture load was determined from the peak load on the load-displacement curve. The specimens were observed under an optical microscope. The fracture modes were classified into three types: cohesive failure within the material (Type-I), adhesive failure (Type-II), and mixed failure with both interfacial debonding and cohesive failure within the material (Type-III) (29).

#### 2.2.3 Microleakage test

Specimen preparation was similar to the fracture test. Reattached roots (*n* = 10) were coated with nail polish, stopping 1 mm from the material's borders. The specimens were immersed in 2% methylene blue solution for 7 days at room temperature. Stained roots were washed and cut horizontally at 1, 6.5, and 13 mm from the coronal side, to obtain three cross-sections per root. The penetration depth along buccal and lingual fracture lines was measured at the three levels using a stereomicroscope (M320, Leica Microsystems, Germany) at 32× magnification.

### 2.3 Statistical analysis

Statistical analyses were conducted using GraphPad Prism version 9.5.1 (GraphPad Software, San Diego, CA, USA). The Shapiro–Wilk tests and Brown-Forsythe test were employed to examine normality and homogeneity of the data before statistical comparison. One-way ANOVA was performed followed by Tukey's multiple-comparisons test. The significance level was set at *α* = 0.05.

## 3 Results

### 3.1 Biocompatibility

#### 3.1.1 CCK-8

**Figure 1:**
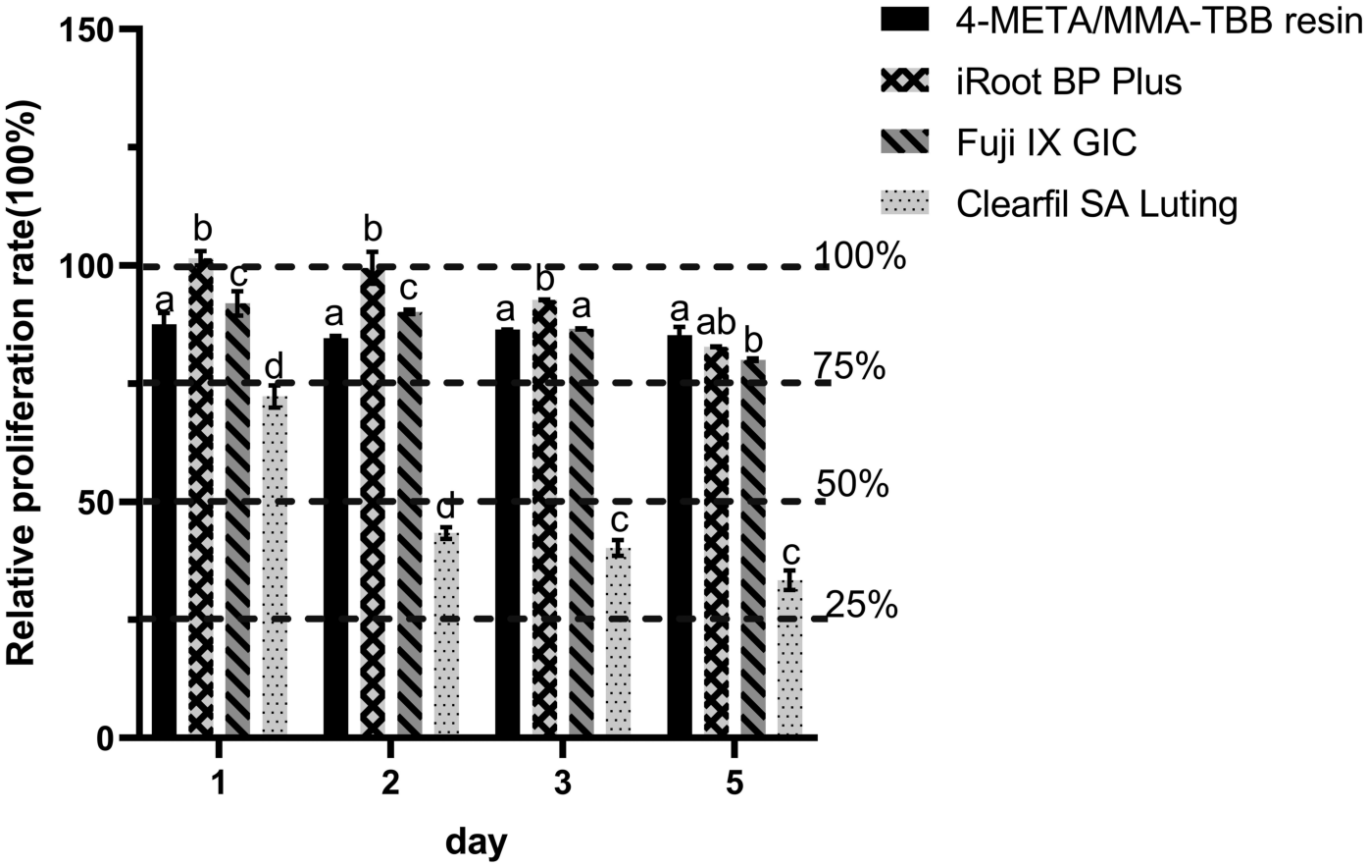
Relative growth ratio of L929 fibroblasts after co-culturing with different extracts for 1, 2, 3, and 5 days. Dotted lines are set to visualize the cytotoxicity classification. different letters indicate significant differences between groups. (*P* < 0.05).

As shown in Figure 1, the cytotoxicity of 4-META/MMA-TBB resin, iRoot BP Plus, and Fuji IX GIC was acceptable, categorized as Grade 0–1 (above 75%) on all the test days. Clearfil SA Luting showed mild cytotoxicity (Grade 2) on day 1 and moderate cytotoxicity (Grade 3) on days 2, 3, 5. The RGR in the iRoot BP Plus group were significantly higher on day 1, but decreased with time and presented no statistical difference with the 4-META/MMA-TBB resin or Fuji IX GIC on day 5.

#### 3.1.2 Live/dead cell staining

**Figure 2:**
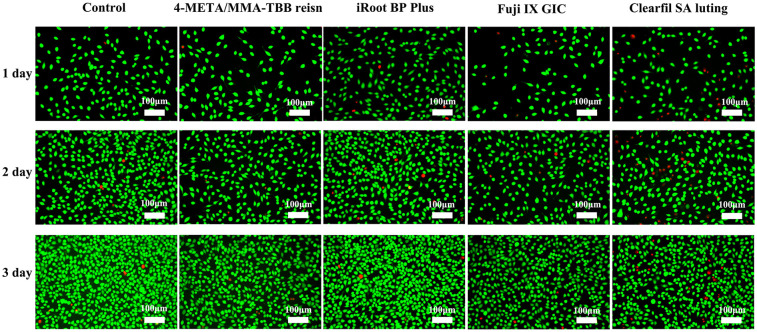
Fluorescence microscopy images of live/dead stained L929 at 1, 2, and 3 days. The green fluorescence represents live cells while the red fluorescence represents live cells (×50).

Live cells exhibited green fluorescence, and dead cells were stained red. Along with culture exposure to the extracts, the cell morphology changed from fusiform or polygonal to round, and the number of dead cells increased. This was most noticeable in the Clearfil SA Luting group (Figure 2).

#### 3.1.3 Flow cytometry

**Figure 3:**
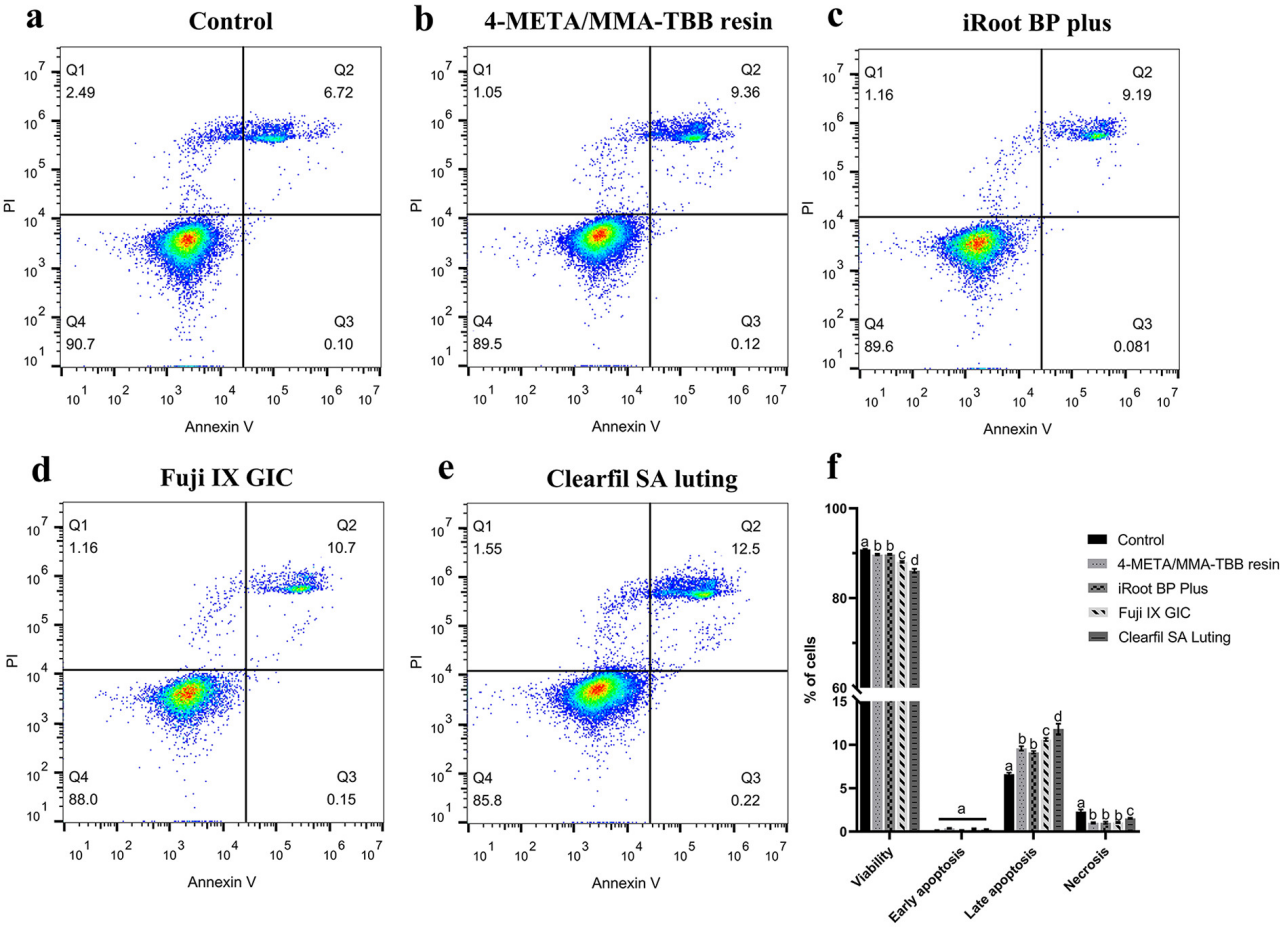
Flow cytometry analysis of mouse L929 fibroblasts treated with different materials. **(a**–**e)** Dot plots representing the distribution of viable (Annexin V^−/^PI^−^), early-apoptotic (Annexin V^+^/PI^−^), late-apoptotic (Annexin V^+^/PI^+^), and necrotic (Annexin V^−^/PI^+^) cells, respectively. **(f)** A histogram comparing the proportions of cell at different stages. The results show Mean ± SD of 3 parallel experiments performed in triplicate. Different letters indicate significant differences between groups. (*P* < 0.05).

Figure 3 illustrates the distribution of viable (Annexin V^−^/PI^−^), early-apoptotic (Annexin V^+^/PI^−^), late-apoptotic (Annexin V^+^/PI^+^), and necrotic (Annexin V^−^/PI^+^) cells. The proportion of viable cells in iRoot BP Plus and 4-META/MMA-TBB resin groups was similar (*P* > 0.05), only lower than that of the control group (*P* < 0.05). Clearfil SA Luting group exhibited the lowest proportion of viable cells, whereas Fuji IX GIC showed a middle proportion value (*P* < 0.05). The proportion of late apoptotic-cells showed an opposite trend among different groups.

### 3.2 Fracture resistance and fracture patterns

As shown in Table 3, there were no significant differences in the buccolingual or mesiodistal diameters among groups (*P* > 0.05). The highest fracture resistance was obtained in sound roots (control) as 278.50 ± 45.19 N, while the lowest value was observed in roots reattached with the Fuji IX GIC as 53.16 ± 15.76 N (*P* < 0.05). The difference between the 4-META/MMA-TBB resin group (220.90 ± 45.01 N) and the Clearfil SA Luting group (184.00 ± 45.45 N) was not statistically significant (*P* > 0.05).

**Figure 4:**
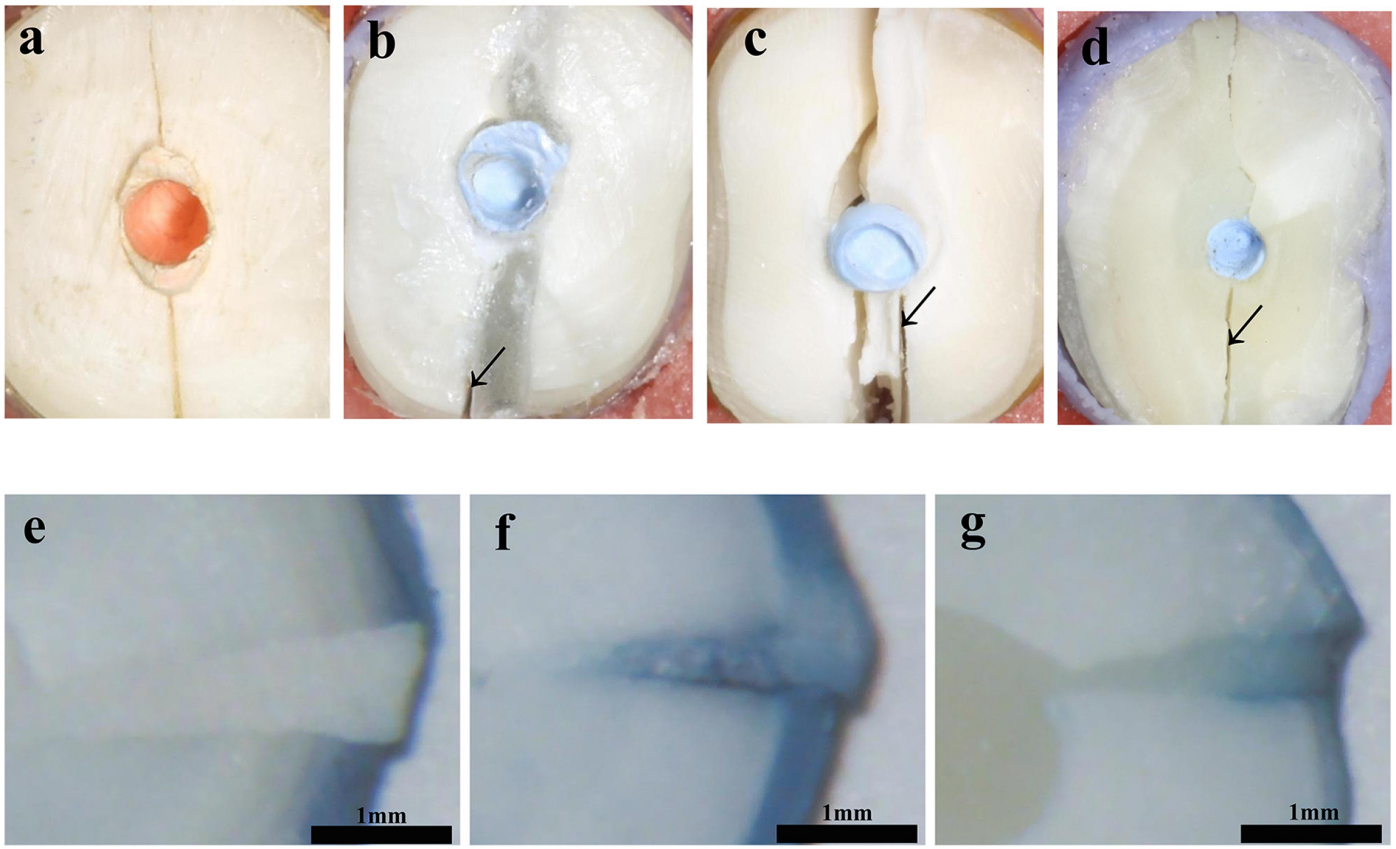
Cross sections of specimens after fracture loading and microleakage of reattached roots (×32): **(a)** Control; **(b,e)** 4-META/MMA-TBB resin; **(c,f)** Fuji IX GIC; **(d,g)** Clearfil SA Luting.

Sound roots (control) were fractured mainly in the buccolingual direction. The fracture patterns of reattached roots were mainly characterized by type-III mixed fractures. More areas of interfacial debonding were observed in the two resin groups, while more cohesive failure areas with irregular initiation and propagation of cracks were observed in the Fuji IX GIC group. Type-II adhesive failure was also observed in two 4-META/MMA-TBB resin reattached specimens and one Clearfil SA Luting reattached specimen. There was no type-I fracture (Figure 4).

### 3.3 Microleakage

Table 3 and Figure 4 compared the microleakage in different groups. The 4-META/MMA-TBB resin group had the shortest penetration depth of only 0.628 ± 0.150 mm, while the Fuji IX GIC group showed the highest value of 2.083 ± 0.170 mm (*P* < 0.05).

## 4 Discussion

Recent advancements indicate the potential of reattaching the fractured fragments in management of VRF teeth (6, 8). The treatment would mostly depend on the biocompatibility and bonding strength of the adhesive materials. In the present study, the biocompatibility of 4-META/MMA-TBB resin was evaluated in comparison with iRoot BP Plus, an excellent and widely used root repair material (7). The fracture resistance and microleakage of reattached roots, were compared with materials commonly used in VRF management, except for iRoot BP Plus for its weak bonding strength. Bovine teeth were selected for the fracture and microleakage tests due to the high availability to standardize age and root size, and their oval roots resemble those susceptible to VRF (30, 31).

The results of the biocompatibility tests indicated that the 4-META/MMA-TBB resin exhibits acceptable biocompatibility towards L929 fibroblasts, which was similar to that of iRoot BP Plus (Figures 1–3). Previous research also found that 4-META/MMA-TBB resin has good biocompatibility to be used as root-end-filling material, compared with MTA, another calcium silicate-based cement. It could promote the adhesion, proliferation, and matrix layer formation of MC3T3-E1 cells, which might enhance apical tissue regeneration (32). CCK-8 in the present study showed that the RGRs of the 4-META/MMA-TBB resin group were lower than those of iRoot BP Plus on the first 3 days, but the difference was insignificant on day 5 (*P* > 0.05). However, Clearfil SA Luting, also a resin, showed the lowest biocompatibility in all the 3 tests. This might be attributed to the free monomers in the case of incomplete polymerization. These free monomers have strong chemical activity and will react with biological macromolecules such as proteins and nucleic acids in the cells, damaging the cell structure and function, thus severely inhibiting the growth and survival of the cells (33). The amount of accumulated free monomers increases gradually with time, which will continuously damage the cell structure and affect the cell biocompatibility (15). This can be seen from the decrease of RGR in Clearfil SA Luting group with time. 4-META/MMA-TBB has a unique catalyst TBB, which was reported to accelerate the polymerization process upon contact with water and air (34). The degree of polymerization of MMA and 4-META monomers could reach up to 82% in 30 min after initiation, compared with 66% of traditional resin cements, and the polymerization continues leaving less unpolymerized monomers (21, 35). These enable 4-META/MMA-TBB resin to be used in root repair with good biosafety.

In CCK-8 assay, iRoot BP Plus caused a decrease in cell biocompatibility over time. The biocompatibility of calcium silicate-based materials may come from the large amount of calcium hydroxide generated during the initial curing process, creating a highly alkaline environment that leads to cell apoptosis (36). The tantalum pentoxide, an inhibitor in iRoot BP Plus, was also found to reduce the biocompatibility (37). The cell biocompatibility of GIC is moderate from the flow cytometry analysis. The cytotoxic effects of GIC was reported from the acidic components, such as tartaric acid, itaconic acid or polyacrylic acid (38), and low concentrations of ions such as F, Al^3+^, SR^2+^, Zn^2+^ (39).

According to the results of the fracture test, sound roots (control) showed the highest fracture resistance (*P* < 0.05). The fractures mainly occurred in the buccolingual direction (Figure 4a), which is consistent with the fracture lines of VRFs (40). Regarding the reattached roots, both the 4-META/MMA-TBB resin group and the Clearfil SA Luting group exhibited relatively high fracture resistance, which could reach 79.3% and 66.1% that of intact teeth, respectively. Previous literature has reported that there was no statistically significant difference in fracture load between the reattached VRF roots bonded with 4-META/MMA-TBB resin and the sound roots (10). The difference between studies may be caused by factors such as the varying strengths of selected teeth and the experimental methods. In our study, 7-year-old bovine teeth were selected to simulate the situation that VRF is prone to occur in the elderly (31, 41). This might cause the mechanical properties of sound roots in the present study to be different from that in other studies (41). Moreover, the reattaching method, such as filling the root canal with gutta-percha or adhesive materials, differed among studies (10, 26) and might affect the fracture resistance of the reattached roots. This deserves further research to guide clinical operations. From the Type-III mixed fracture pattern of two resin cement groups, more failures were presented at the bonding interface than within the materials (Figures 4b,d), suggesting that the bonding interface is still the weak point of the reattached roots (42).

The bonding advantage of 4-META/MMA-TBB resin over Clearfil SA Luting was not statistically significant in the present study. But it has been reported that VRF roots attached with 4-META/MMA-TBB resin showed higher fracture resistance than that of a self-adhesive dual cure resin (26). In clinical settings, when performing bonding and replantation for roots affected by VRF within the limited time, the bonding surface may be contaminated with blood and not be removed completely. 4-META/MMA-TBB resin was reported to have unaffected bond strength when used on blood contaminated dentin, because of its pretreatment with the 10% citric acid +3% FeCl_3_ solution (22). It can also polymerize within the extraction socket, and does not require a long polymerization process like dual-cure resin (8). These makes 4-META/MMA-TBB resin more suitable for practical clinical applications.

Fuji IX GIC provided the lowest fracture force, which could be interpreted by the bonding mechanisms as well as its low material strength. GIC achieves weak bonding effect by forming ionic bonds with calcium ions of the hydroxyapatite in the tooth structure (43). But resin cements gains excellent bonding from the hybrid layer formed by the penetration of resin monomers into the demineralized collagen fibers (9). Moreover, the low material strength of Fuji IX GIC might be the reason of cohesive failure within the material (Figure 4c), and this may be related to the large glass particles and voids present in the material (44, 45). Resin materials are well known for their excellent mechanical properties, especially 4-META/MMA-TBB resin, which is a pure resin adhesive without any fillers and has good compressive strength and ductility (46).

From the results of microleakage, 4-META/MMA-TBB resin exhibited the best sealing capability to prevent the penetration of liquids and bacteria through the interface effectively (47). As a functional monomer, 4-META can form strong chemical bonds with the tooth structure, and promote the infiltration of MMA monomers into the dentin and combine with groups such as hydroxyl groups in the dentin, which tightly connects the resin and the tooth. In addition, MMA is a small molecule with high diffusivity, enabling the resin to better conform to the irregular shape of the tooth surface, filling the tiny gaps and depressions (48), and enhancing the marginal sealing performance greatly. 4-META/MMA-TBB resin could penetrate the entire demineralized portion of dentin etched with 10% citric acid + 3% FeCl_3_ solution for 10 s and reach the underlying intact dentin before polymerization initiation (49). It's worth noting that the microleakage distance increased with the prolonged etching time after 10s (50). In this study, the etching time was controlled at 10s. Clearfil SA Luting was reported to undergo phototropic polymerization in the early stage, which formed gaps in the resin infiltration layer, resulting in poor marginal sealing and the decreased bonding effect as well (51). Fuji IX GIC is known to bond chemically to tooth structure, but it exhibits poor microleakage performance. Glass ionomer cement was extremely sensitive to humidity due to water absorption and the hydrolysis of the cement matrix even after curing. This impaired the edge integrity and lead to microleakage (52–54).

Taking all the above results into consideration, the 4-META/MMA-TBB resin demonstrated a L929 fibroblasts biocompatibility similar to that of iRoot BP Plus, suggesting its applicability for root repair. Used for reattachment of VRF affected roots, 4-META/MMA-TBB resin provided adequate fracture resistance to bear a certain magnitude of masticatory forces and offered outstanding sealing effect to prevent microleakage. This provides beneficial insights into the potential of 4-META/MMA-TBB resin in the reattachment of fractured roots, endeavor to preserve nature teeth affected by VRFs. Though Clearfil SA Luting provides moderate fracture resistance and microleakage, their biocompatibility limits their application in treatment. The biocompatibility of Fuji IX GIC is acceptable, but reattached roots showed lowest fracture resistance and poor microleakage. Nonetheless, further studies are needed to explore the *in vivo* healing of periodontal tissues after reattaching of the fractured roots.

## 5 Conclusions

Within the limitations of this study, the following conclusions were reached:

1. 4-META/MMA-TBB resin had acceptable cell biocompatibility for root repair, similar to that of iRoot BP Plus.
2. It can provide adequate fracture resistance and outstanding sealing effect for reattaching treatment of VRFs.

## Data Availability

The original contributions presented in the study are included in the article/Supplementary Material, further inquiries can be directed to the corresponding author.
